# Patterns of medicinal cannabis use, strain analysis, and substitution effect among patients with migraine, headache, arthritis, and chronic pain in a medicinal cannabis cohort

**DOI:** 10.1186/s10194-018-0862-2

**Published:** 2018-05-24

**Authors:** Eric P. Baron, Philippe Lucas, Joshua Eades, Olivia Hogue

**Affiliations:** 1 Center for Neurological Restoration - Headache and Chronic Pain Medicine, Department of Neurology, Cleveland Clinic Neurological Institute, 10524 Euclid Avenue, C21, Cleveland, OH 44195 USA; 2 Tilray, 1100 Maughan Rd, Nanaimo, BC V9X 1J2 Canada; 3 Social Dimensions of Health, University of Victoria, 3800 Finnerty Rd, Victoria, BC V8P 5C2 Canada; 4 Canadian Institute for Substance Use Research, 2300 McKenzie Ave, Victoria, BC V8N 5M8 Canada; 5 Section of Biostatistics, Department of Quantitative Health Sciences, Cleveland Clinic Lerner Research Institute, 9500 Euclid Avenue, JJN3, Cleveland, OH 44195 USA

**Keywords:** Cannabis, Cannabinoids, Marijuana, CBD, Cannabidiol, THC, Δ^9^-tetrahydrocannabinol, Migraine, Headache, Terpenes, Arthritis, Pain

## Abstract

**Background:**

Medicinal cannabis registries typically report pain as the most common reason for use. It would be clinically useful to identify patterns of cannabis treatment in migraine and headache, as compared to arthritis and chronic pain, and to analyze preferred cannabis strains, biochemical profiles, and prescription medication substitutions with cannabis.

**Methods:**

Via electronic survey in medicinal cannabis patients with headache, arthritis, and chronic pain, demographics and patterns of cannabis use including methods, frequency, quantity, preferred strains, cannabinoid and terpene profiles, and prescription substitutions were recorded. Cannabis use for migraine among headache patients was assessed via the ID Migraine™ questionnaire, a validated screen used to predict the probability of migraine.

**Results:**

Of 2032 patients, 21 illnesses were treated with cannabis. Pain syndromes accounted for 42.4% (*n* = 861) overall; chronic pain 29.4% (*n* = 598;), arthritis 9.3% (*n* = 188), and headache 3.7% (*n* = 75;). Across all 21 illnesses, headache was a symptom treated with cannabis in 24.9% (*n* = 505). These patients were given the ID Migraine™ questionnaire, with 68% (*n* = 343) giving 3 “Yes” responses, 20% (*n* = 102) giving 2 “Yes” responses (97% and 93% probability of migraine, respectively). Therefore, 88% (*n* = 445) of headache patients were treating probable migraine with cannabis. Hybrid strains were most preferred across all pain subtypes, with “OG Shark” the most preferred strain in the ID Migraine™ and headache groups. Many pain patients substituted prescription medications with cannabis (41.2–59.5%), most commonly opiates/opioids (40.5–72.8%). Prescription substitution in headache patients included opiates/opioids (43.4%), anti-depressant/anti-anxiety (39%), NSAIDs (21%), triptans (8.1%), anti-convulsants (7.7%), muscle relaxers (7%), ergots (0.4%).

**Conclusions:**

Chronic pain was the most common reason for cannabis use, consistent with most registries. The majority of headache patients treating with cannabis were positive for migraine. Hybrid strains were preferred in ID Migraine™, headache, and most pain groups, with “OG Shark”, a high THC (Δ9-tetrahydrocannabinol)/THCA (tetrahydrocannabinolic acid), low CBD (cannabidiol)/CBDA (cannabidiolic acid), strain with predominant terpenes β-caryophyllene and β-myrcene, most preferred in the headache and ID Migraine™ groups. This could reflect the potent analgesic, anti-inflammatory, and anti-emetic properties of THC, with anti-inflammatory and analgesic properties of β-caryophyllene and β-myrcene. Opiates/opioids were most commonly substituted with cannabis. Prospective studies are needed, but results may provide early insight into optimizing crossbred cannabis strains, synergistic biochemical profiles, dosing, and patterns of use in the treatment of headache, migraine, and chronic pain syndromes.

## Background

The legal use of medicinal cannabis continues to increase globally, including the United States. At the time of this writing, there are currently 29 states which have legalized medicinal cannabis, 9 states and Washington DC which have legalized both medicinal and recreational cannabis use, and 18 states which have legalized cannabidiol (CBD)-only bills.

The use of medicinal cannabis for a multitude of health maladies, particularly chronic pain, has been well described through ancient, historical, and current times, and well supported through the medical literature [1–28]. In 2017, The National Academies of Sciences, Engineering, and Medicine published a statement that the use of cannabis for the treatment of pain is supported by well-controlled clinical trials and that there is substantial evidence that cannabis is an effective treatment for chronic pain in adults [24]. In 2014, the Canadian Pain Society revised their consensus statement to recommend cannabinoids as a third-level therapy for chronic neuropathic pain given the evidence of cannabinoid efficacy in the treatment of pain with a combined number needed to treat (NNT) of 3.4 [25]. Most medicinal cannabis registries report that chronic pain is the most common indication for use [29–39]. However, most of these registries do not further differentiate chronic pain into different pain subsets.

Supporting evidence also exists for cannabis/cannabinoids in the treatment of migraine and/or chronic migraine [1, 40–56], cluster headache [56–59], chronic headaches [13, 44, 60, 61], medication overuse headache [62], idiopathic intracranial hypertension [63], and multiple sclerosis associated trigeminal neuralgia [64]. Publications detailing this headache, migraine, and facial pain literature, as well as described mechanisms of pain relief with cannabis and cannabinoids are available and should be reviewed, but are beyond the scope of this paper [1, 2, 28, 51, 65]. At the time of this writing, the limited supporting headache literature consists of one retrospective analysis, numerous case series, case studies, and case reports, clinical/anecdotal reports, and surveys. There are no placebo-controlled studies of cannabis for headache disorders, although a multicenter, double-blind, placebo-controlled study evaluating efficacy and safety of a synthetic Δ^9^-tetrahydrocannabinol (THC), Dronabinol, in a metered dose inhaler for the treatment of migraine with and without aura has been completed, but results not available [66]. There are only two prospective trials containing a control group evaluating the use of cannabinoids in the treatment of headache disorders, specifically chronic migraine, cluster headache, and medication overuse headache [56, 62].

The first of these two prospective trials was a randomized, double-blind, active-controlled crossover trial with treatment refractory medication overuse headache (MOH) with daily analgesic intake for at least 5 years and several failed detoxification attempts. Patients completed a course of either Ibuprofen 400 mg or Nabilone 0.5 mg daily for 8 weeks, had a 1 week washout, then a second 8 weeks of the other medication. Results showed that Nabilone 0.5 mg daily, a synthetic cannabinoid, was superior in reducing daily analgesic intake, pain intensity, level of medication dependence, and improved quality of life in these patients [62].

The second prospective trial evaluated the use of cannabinoids as both a prophylaxis and acute treatment for both chronic migraine and chronic cluster headache [56]. Patients were given one of two compounds containing 19% THC or a combination of 0.4% THC + 9% CBD. In phase 1, dose finding observations to determine effective dosing was performed with a group of 48 chronic migraineurs. It was found that doses less than 100 mg produced no benefit, while an oral dose of 200 mg administered during a migraine attack decreased acute pain intensity by 55%, which was the dose used in phase 2. In phase 2, chronic migraine patients were assigned to 3 months prophylaxis treatment with either 25 mg per day of Amitriptyline or THC + CBD 200 mg per day. Chronic cluster headache patients were assigned to 1 month prophylaxis treatment with either Verapamil 480 mg per day or THC + CBD 200 mg per day. For acute pain attacks, additional dosing of THC + CBD 200 mg was allowed in both groups. In the migraine patients, the THC + CBD 200 mg prophylaxis provided a 40.4% improvement versus 40.1% with Amitriptyline. In the cluster headache patients, the THC + CBD 200 mg prophylaxis gave minimal to no benefit. Additional acute THC + CBD 200 mg dosing decreased pain intensity in migraine patients by 43.5%. This same result was seen in cluster headache patients, but only if they had a history of migraine in childhood. In cluster headache patients without a previous history of childhood migraine, the additional THC-CBD 200 mg abortive treatment provided no benefit as an acute treatment.

It is unclear whether certain types of pain may respond better to certain cannabis strains with specific combinations of cannabinoids, terpenes, or other biochemical properties. There have been a multitude of studies showing benefit in many forms of chronic pain, but there have been no studies attempting to differentiate which types and strains of cannabis along with associated compositions of cannabinoids and terpenes may be more effective for certain subsets of pain. This information would be of great clinical use in providing direction for treatment recommendations by healthcare providers.

## Methods

Appropriate Investigational Review Boards approved the survey. A French and English electronic survey was sent to 16,675 Tilray medicinal cannabis patients. Tilray is a federally authorized medical cannabis production, distribution, and research company in Nanaimo, British Columbia. Data gathering was performed with REDCap (Research Electronic Data Capture), a HIPAA and PIPEDA compliant secure web application for building and managing online surveys and databases. A $10 account credit was offered to each patient completing the online survey, funded by Tilray. There was a response of 3405 (3390 English and 15 French), 2032 of which provided a verifiable Tilray patient number and were therefore included in the final analysis. The responses represent 12% of those reached. Recruitment was deliberately halted at 2000 (overlap with additional 32 subjects represents participants who were in the middle of completing the survey when it was halted). The survey launched at 9 AM PST on Monday January 9th 2017 and closed on Wednesday January 11th 2017 at 5 PM PST. The limit to responses was due to financial constraints, and patients were informed that the survey would be available for a two-week period or until limit was reached, whichever came first.

An estimation of migraine prevalence among those surveyed was obtained by incorporating the ID Migraine™ questionnaire [67] into the survey, which is used to predict the probability of migraine. In the ID Migraine™ questionnaire, the patient is given 3 questions. If the patient answers “Yes” to 3 of these questions, there is a 97% chance they have migraine. If they answer “Yes” to 2 of these questions, there is a 93% chance they have migraine. The questions are: 1) Have your headaches interfered with your ability to work, study, or do what you needed to do? 2) Have you felt nauseated or sick to your stomach when you have a headache? 3) Does light bother you when you have a headache (a lot more than when you don’t have a headache)?

Patients were asked a multitude of additional questions involving demographics, primary illnesses and symptoms treated with cannabis, frequency and quantity of use, favorite cannabis types and strains, methods of use, and prescription drugs substituted with cannabis.

Patients who reported headache as the primary illness were compared with each patient group reporting a diagnosis other than headache as the primary illness. Separately, patients who reported headache as the primary symptom (regardless of diagnosis) were compared with each patient group who both reported a diagnosis other than headache as the primary illness and also did not report headache as the primary symptom. Statistical methods were the same for each set of comparisons. Pearson chi-squared tests, or Fisher’s exact tests where appropriate, were used to compare headache patients with each non-headache patient group, with regards to five cannabis strains: Hybrid, Indica, Sativa, 3:1 CBD:THC, and 1:1 CBD:THC. Significance for omnibus chi-squared tests was designated by *p* < .05. When omnibus chi-squared tests were found to be significant, pairwise comparisons were carried out using a Bonferroni correction. Given ten pairwise comparisons per omnibus test, significance for each pairwise comparison was indicated by *p* < .005. Methods chosen to control for multiple comparisons allow a moderately conservative level of control, and reflect the exploratory nature of the study. Analyses were two-tailed and performed using SAS Studio v 3.5.

## Results

Of the 2032 patients included in the survey, 1271 (62.6%) were male, 758 (37.3%) were female, and 3 (0.15%) did not specify gender. Ages ranged from 9 to 85 years old, with an average age of 40. Reported ethnicities in the overall cohort revealed 1839 (90.5%) Caucasian, 62 (3.1%) Metis, 60 (3%) Aboriginal/First Nation, 39 (1.9%) Other, 37 (1.8%) South Asian (East Indian, Pakistani, Sri Lankan, etc.), 35 (1.7%) Asian (Chinese, Japanese, Korean, Vietnamese, etc.), 25 (1.2%) Black (African, Caribbean, etc.), and 24 (1.2%) Hispanic (Mexican, Central American, South America, etc.), with some patients reporting more than one ethnicity. Relationship status showed 833 (41%) were married, 507 (25%) were single and never married, 274 (13.5%) were in a domestic partnership or civil union, 203 (10%) were single but cohabiting with a significant other, 132 (6.5%) were divorced, 64 (3.2%) were separated, and 19 (0.94%) were widowed. Habitation showed 883 (43.5%) to be living in an urban area, 795 (39.1%) in a suburban area, and 354 (17.4%) in a rural or remote area.

There were 21 primary illnesses that were reported as being treated with medicinal cannabis, as seen in Table 1. The subsets analyzed further were headache, chronic pain, and arthritis. Chronic pain was the most frequently reported primary illness for which medicinal cannabis was being used at 29.4% (*n* = 598), arthritis was 9.3% (*n* = 188), and headache was 3.7% (*n* = 75). Notably, when combined these three categories of pain syndromes accounted for 42.4% (*n* = 861) of the entire medicinal cannabis users.

**Table 1:** Primary illness treated with medicinal cannabis

Primary Illness	Total	Male	Female	Unspecified
*n*	2032	1271 (62.6%)	758 (37.3%)	3 (0.15%)
Chronic Pain	598 (29.4%)	371 (62%)	227 (38%)	
Mental Health Condition	548 (27%)	319 (58.2%)	228 (41.6%)	1 (0.2%)
Insomnia/Sleep Disorder	198 (9.7%)	145 (73.2%)	53 (26.8%)	
Arthritis/Musculoskeletal	188 (9.3%)	112 (59.6%)	76 (40.4%)	
PTSD	93 (4.6%)	59 (63.4%)	33 (35.5%)	1 (1.1%)
Headache	75 (3.7%)	44 (58.7%)	31 (41.3)	
Gastrointestinal Disorder	62 (3.1%)	34 (54.8%)	28 (45.2%)	
Multiple sclerosis	45 (2.2%)	26 (57.8%)	19 (42.2%)	
Other	38 (1.9%)	23 (60.5%)	15 (39.5%)	
Cancer/Leukemia	35 (1.7%)	24 (68.6%)	11 (31.4%)	
Crohn’s Disease	35 (1.7%)	27 (77.1%)	8 (22.9%)	
Brain Injury	24 (1.3%)	16 (66.7%)	8 (33.3%)	
Epilepsy/Seizure Disorder	21 (1.0%)	18 (85.7%)	3 (14.3%)	
Eating Disorder	20 (1.0%)	10 (50%)	10 (50%)	
Diabetes	16 (0.79%)	13 (81.3%)	3 (18.7%)	
Movement Disorder	10 (0.49%)	8 (80%)	1 (10%)	1 (10%)
AIDS/HIV	8 (0.39%)	7 (87.5%)	1 (12.5%)	
Hepatitis	6 (0.30%)	6 (100%)	0 (0%)	
Glaucoma	5 (0.25%)	5 (100%)	0 (0%)	
Osteoporosis	4 (0.20%)	3 (75%)	1 (25%)	
Skin Condition	3 (0.15%)	1 (33.3%)	2 (66.7%)	

Headache was then evaluated as a primary symptom being treated by medicinal cannabis across all primary illnesses (headache was the major symptom being treated with medicinal cannabis, among the primary illness categories), as seen in Table 2. There were 505 patients within the entire group surveyed (24.9%) who reported headache as a primary symptom for which they were using medicinal cannabis across all primary illness categories. Of these patients, 262 (51.9%) were male, 241 (47.7%) were female, and 2 (0.40%) did not specify gender. Ages ranged from 10 to 86 years old with an average age of 38. Reported ethnicities revealed 453 (89.7%) Caucasian, 23 (4.6%) Metis, 21 (4.2%) Aboriginal/First Nation, 12 (2.4%) Other, 11 (2.2%) Hispanic (Mexican, Central American, South America, etc.), 10 (2%) Asian (Chinese, Japanese, Korean, Vietnamese, etc.), 8 (1.6%) South Asian (East Indian, Pakistani, Sri Lankan, etc.), and 4 (0.8%) Black (African, Caribbean, etc.), with many patients reporting more than one ethnicity. Relationship status showed 181 (36%) were married, 125 (24.8%) were single and never married, 88 (17.4%) were in a domestic partnership or civil union, 62 (12.3%) were single but cohabiting with a significant other, 28 (5.5%) were divorced, 18 (3.6%) were separated, and 3 (0.6%) were widowed. Habitation showed 218 (43.2%) to be living in an urban area, 205 (40.6%) in a suburban area, and 82 (16.2%) in a rural or remote area. Chronic pain was the most common primary illness in which headache was reported to be a primary symptom being treated with medicinal cannabis (29.3%), followed by mental health condition (25.9%) and headache (14.9%).

**Table 2:** Headache as primary symptom treated with medicinal cannabis among various primary illnesses reported

Primary Illness	Total	Male	Female	Unspecified
*n*	505	262 (51.9%)	241 (47.7%)	2 (0.40%)
Chronic pain	148 (29.3%)	70 (47.3%)	78 (52.7%)	
Mental Health Condition	131 (25.9%)	65 (49.6%)	66 (50.4%)	
Headache	75 (14.9%)	44 (58.7%)	31 (41.3%)	
Insomnia	32 (6.3%)	25 (78.1%)	7 (21.9%)	
Arthritis/Musculoskeletal	29 (5.7%)	12 (41.4%)	17 (58.6%)	
PTSD	24 (4.8%)	9 (37.5%)	14 (58.3%)	1 (4.2%)
MS	13 (2.6%)	3 (23.1%)	10 (76.9%)	
Brain Injury	12 (2.4%)	8 (66.7%)	4 (33.3%)	
Gastrointestinal Disorder	11 (2.2%)	5 (45.5%)	6 (54.5%)	
Cancer/Leukemia	6 (1.2%)	3 (50%)	3 (50%)	
Movement Disorder	5 (1.0%)	4 (80%)	0 (0%)	1 (20%)
Other	4 (0.79%)	2 (50%)	2 (50%)	
Epilepsy/Seizure Disorder	3 (0.59%)	2 (66.7%)	1 (33.3%)	
Crohn’s Disease	3 (0.59%)	3 (100%)	0 (0%)	
Diabetes	2 (0.40%)	1 (50%)	1 (50%)	
Glaucoma	2 (0.40%)	2 (100%)	0 (0%)	
Hepatitis	2 (0.40%)	2 (100%)	0 (0%)	
Eating Disorder	1 (0.20%)	1 (100%)	0 (0%)	
AIDS/HIV	1 (0.20%)	1 (100%)	0 (0%)	
Osteoporosis	1 (0.20%)	0 (0%)	1 (100%)	

The 505 patients who reported headache as a primary symptom being treated by medicinal cannabis were then analyzed to estimate how many of those patients had probable migraine, and thus, how many were using medicinal cannabis for probable migraine management. This data was obtained via responses to the ID Migraine™ questionnaire. There were 343 (68%) who gave 3 “Yes” responses, and 102 (20%) who gave 2 “Yes” responses. Based on these responses, 445 of these 505 patients (88%) had a very high probability between 93 and 97% that the headaches they were treating with medicinal cannabis represented migraine.

Data was collected among patients to determine the most commonly used and preferred types of cannabis, as well as preferred specific strains. The preferred types of cannabis included Indica, Sativa, Hybrid, 3:1 CBD:THC, or 1:1 CBD:THC. Indicas, Sativas and Hybrids were all high THC/low CBD strains or extracts, while 1:1 and 3:1 strains and extracts represent the CBD:THC ratio, and were considered high CBD strains. The Indica, Sativa, and Hybrid types were further divided into specific strains within each of these cannabis types.

There were 42 different preferred treatment strains reported by patients and these included: Afghani, Afghani CBD, Alien OG, Barbara Bud, Black Tuna, Blueberry, Bubba Kush, Cannatonic, CBD House Blend, Cheese, Churchill, Dig Weed, Elwyn, Green Cush, Girl Scout Cookies (GSC), Harmony, Headband, Hybrid House Blend, Indica House Blend, Island Sweet Skunk, Jack Herer, Jean Guy, Lemon Sour Diesel, Limonene House Blend, Mango, Master Kush, Myrcene Blend, OG Kush, OG Shark, Pinene House Blend, Pink Kush, Purple Kush, Rockstar, Sativa House Blend, Sirius, Strawberry Cough (SBC), Skywalker OG, Sour Diesel, Sweet Skunk CBD, Warlock CBD, Watermelon, and White Widow.

Preferred cannabis types and strains were first analyzed between the headache as primary symptom, headache as primary illness, chronic pain as primary illness, and arthritis as primary illness groups. Hybrid strains were the most commonly preferred cannabis types across all pain groups. However, when patients with headache as a primary symptom were excluded from the groups, the arthritis group preferred Indica strains, while the others still preferred Hybrid strains. The top 15 preferred cannabis strains within each of these pain groups are seen in Tables 3 and 5. Preferred cannabis types and strains were then analyzed in the positive ID Migraine™ patients who answered 3 “Yes” responses (343), 2 “Yes” responses (102), or combined 3 + 2 “Yes” responses (445) to the ID Migraine™ questionnaire. Thus, they were the most probable group of headache patients who were treating migraine with medicinal cannabis. Hybrid strains were the most commonly preferred cannabis types across the positive ID Migraine™ groups with the exception that the 2 “Yes” group had a slight preference for Sativa, followed by Hybrid strains. The top 15 preferred cannabis strains within each positive ID Migraine™ group are seen in Table 3. “OG Shark” was the most commonly preferred strain across all of the positive ID Migraine™ and headache as primary symptom groups. Quantification and comparison of the cannabinoids and terpenes present in these top 15 preferred strains is seen in Table 4. The cannabinoids analyzed were Δ^9^-tetrahydrocannabinol (THC), tetrahydrocannabinolic acid (THCA), cannabidiol (CBD), and cannabidiolic acid (CBDA). The terpenes analyzed were α-pinene, β-myrcene, D-limonene, linalool, β-caryophyllene, humulene, trans-nerolidol, and bisabolol. Notably, “OG Shark”, a high THC/THCA, low CBD/CBDA strain with β-caryophyllene followed by β-myrcene as the predominant terpenes, was the most preferred strain in both the positive ID Migraine™ and headache as primary symptom groups.

**Table 3:** Preferred medicinal cannabis types and strains among headache patients and probable migraineurs based on “Yes” responses on ID Migraine™ questionnaire

Preferred Cannabis Type					
	Headache as primary illness (75)	3 Yes^a^ (343)	2 Yes^b^ (102)	3 + 2 Yes (445)	Headache as primary symptom (505)
Hybrid	26 (34.7%)	118 (34.4%)	35 (34.3%)	153 (34.4%)	165 (32.7%)
Indica	19 (25.3%)	106 (30.9%)	20 (19.6%)	126 (28.3%)	144 (28.5%)
Sativa	20 (26.7%)	76 (22.2%)	36 (35.3%)	112 (25.2%)	136 (26.9%)
3:1 CBD:THC	5 (6.7%)	22 (6.4%)	7 (6.9%)	29 (6.5%)	34 (6.7%)
1:1 CBD:THC	5 (6.7%)	20 (5.8%)	4 (3.9%)	24 (5.4%)	25 (5%)
No response	0 (0%)	1 (0.3%)	0 (0%)	1 (0.2%)	1 (0.2%)
Preferred Cannabis Strains (Top 15)					
	Headache as primary illness	3 Yes	2 Yes	3 + 2 Yes	Headache as primary symptom
	Skywalker OG (7; 10.6%)	OG Shark (20; 8.4%)	OG Shark (9; 11%)	OG Shark (29; 8.9%)	OG Shark (34; 9.6%)
	Headband (5; 7.6%)	Afghani (19; 8.0%)	Skywalker OG (8; 9.8%)	Afghani (25; 7.7%)	Jean Guy (29; 8.2%)
	Cannatonic (5; 7.6%)	Jack Herer (19; 8.0%)	White Widow (8; 9.8%)	Skywalker OG (25; 7.7%)	Skywalker OG (28; 7.9%)
	Jack Herer (5; 7.6%)	Jean Guy (19; 8.0%)	Lemon Sour Diesel (7; 8.5%)	Lemon Sour Diesel (25; 7.7%)	Lemon Sour Diesel (28; 7.9%)
	Afghani (4; 6.1%)	Lemon Sour Diesel (18; 7.6%)	Afghani (6; 7.3%)	Jack Herer (24; 7.3%)	Afghani (26; 7.4%)
	Indica House Blend (4; 6.1%)	Skywalker OG (17; 7.1%)	Pink Kush (6; 7.3%)	Jean Guy (24; 7.3%)	White Widow (26; 7.4%)
	Rock Star (4; 6.1%)	Master Kush (16; 6.7%)	Island Sweet Skunk (6; 7.3%)	White Widow (24; 7.3%)	Jack Herer (26; 7.4%)
	Warlock CBD (3; 4.6%)	White Widow (16; 6.7%)	Jack Herer (5; 6.1%)	Pink Kush (21; 6.4%)	Pink Kush (22; 6.2%)
	Sweet Skunk CBD (3; 4.6%)	Sweet Skunk CBD (15; 6.3%)	Jean Guy (5; 6.1%)	Master Kush (20; 6.1%)	Sweet Skunk CBD (21; 5.9%)
	Jean Guy (3; 4.6%)	Pink Kush (15; 6.3%)	Headband (4; 4.9%)	Sweet Skunk CBD (18; 5.5%)	Island Sweet Skunk (21; 5.9%)
	Girl Scout Cookies (GSC) (3; 4.6%)	Headband (13; 5.5%)	Master Kush (4; 4.9%)	Headband (17; 5.2%)	Master Kush (21; 5.9%)
	OG Shark (2; 3%)	Cannatonic (13; 5.5%)	Sour Diesel (4; 4.9%)	Island Sweet Skunk (17; 5.2%)	Black Tuna (20; 5.7%)
	Black Tuna (2; 3%)	Warlock CBD (13; 5.5%)	Black Tuna (4; 4.9%)	Black Tuna (16; 4.9%)	Headband (19; 5.4%)
	Bubba Kush (2; 3%)	Blueberry (13; 5.5%)	Hybrid House Blend (3; 3.7%)	Warlock CBD (14; 4.3%)	Cannatonic (18; 5.1%)
	CBD House Blend (2; 3%), Elwyn (2; 3%), Island Sweet Skunk (2; 3%), Mango (2; 3%), Master Kush (2; 3%), Blueberry (2; 3%), Pink Kush (2; 3%)	Black Tuna (12; 5.0%)	Sweet Skunk CBD (3; 3.7%)	Cannatonic (14; 4.3%), Blueberry (14; 4.3%)	Hybrid House Blend (15; 4.2%)

^a^3 “Yes” responses = 97% probability of migraine

^b^2 “Yes” responses = 93% probability of migraine

**Table 4:** Terpenes and cannabinoids present in top 15 preferred medicinal cannabis strains in headache patients who replied with 3 or 2 “Yes” responses on ID Migraine™ questionnaire

Strain	Terpenes (%)								Cannabinoids (%)			
	α-Pinene	β-Myrcene	D-Limonene	Linalool	β-Caryophyllene	Humulene	Trans-nerolidol	Bisabolol	THCA	THC	CBDA	CBD
OG Shark	0.022	0.194	0.191	0.136	0.263	0.078	0.023	0.107	22.8	21.4	0.1	0
Afghani	0.024	0.101	0.036	0.033	0.132	0.055	0.032	0.066	16.9	15.6	0.1	0
Skywalker OG	0.037	0.217	0.208	0.159	0.319	0.149	0.024	0.110	24.2	22.9	0.2	0
Lemon Sour Diesel	0.127	0.235	0.037	0.026	0.169	0.067	0.022	0.026	19.9	18.3	0.1	0
Jack Herer	0.369	0.612	0.023	0.021	0.132	0.039	0.046	0.013	18.8	17.9	0.2	0
Jean Guy	0.031	0.066	0.069	0.063	0.156	0.047	0.050	0.052	18.1	17.3	0.1	0
White Widow	0.032	0.093	0.195	0.006	0.106	0.032	0.034	0.051	20.1	18.7	0.1	0
Pink Kush	0.019	0.187	0.178	0.148	0.317	0.093	0.058	0.124	27.7	25.8	0.1	0
Master Kush	0.045	0.168	0.192	0.203	0.353	0.169	0.039	0.130	28	25.6	0.1	0
Sweet Skunk CBD	0.054	0.162	0.042	0.014	0.051	0.019	0.015	0.028		9.1		11.2
Headband	0.028	0.238	0.230	0.138	0.318	0.094	0.065	0.124	25.1	23.4	0.1	0
Black Tuna	0.026	0.139	0.149	0.077	0.267	0.088	0.033	0.054	21.8	0.2	0.1	0
Warlock CBD	0.050	0.298	0.199	0.051	0.173	0.102	0.023	0.032	11.4	11	12.6	11.4
Cannatonic	0.059	0.152	0.038	0.022	0.099	0.032	0.015	0.035	10.9	9.4	7.6	7.5
Blueberry	0.000	0.333	0.000	0.052	0.324	0.089	0.021	0.023		21.7		0.1

For further comparison purposes, preferred cannabis types and strains were also analyzed for the three most common non-pain subsets of patients, which included mental health condition/PTSD, insomnia/sleep disorder, gastrointestinal disorder/Crohn’s Disease, and the overall patient cohort, as seen in Table 5. Indica strains were preferred in the insomnia/sleep disorders group, Sativa strains in the mental health condition/PTSD group, and Hybrid strains in the gastrointestinal disorder/Crohn’s Disease group, regardless of whether patients with headache as a primary symptom were included or not. Table 6 shows these same groups, as well as the arthritis and chronic pain groups, with all groups excluding patients with headache as a primary symptom.

**Table 5:** Preferred medicinal cannabis types and strains in all non-headache groups, including patients with headache as primary symptom

Preferred Cannabis Type						
	Chronic pain as primary illness (598)	Arthritis as primary illness (188)	Mental Health Condition (548) /PTSD (93) = (641)	Insomnia/Sleep Disorder (198)	Gastrointestinal Disorder (62) /Crohn’s Disease (35) = (97)	Overall Medicinal Cannabis Cohort (2032)
Hybrid	221 (37%)	57 (30.3%)	177 (27.6%)	61 (30.8%)	37 (38.1%)	651 (32%)
Indica	152 (25.4%)	56 (29.8%)	173 (27%)	88 (44.4%)	16 (16.5%)	569 (28%)
Sativa	121 (20.2%)	34 (18.1%)	207 (32.3%)	39 (19.7%)	23 (23.7%)	502 (24.7%)
3:1 CBD:THC	49 (8.2%)	22 (11.7%)	46 (7.2%)	3 (1.5%)	11 (11.3%)	154 (7.6%)
1:1 CBD:THC	52 (8.7%)	16 (8.5%)	35 (5.5%)	7 (3.5%)	10 (10.3%)	146 (7.2%)
No response	3 (0.5%)	3 (1.6%)	3 (0.5%)	0 (0%)	0 (0%)	10 (0.49%)
Preferred Cannabis Strains (top 15)						
	Chronic pain as primary illness	Arthritis as primary illness	Mental Health Condition/PTSD	Insomnia/Sleep Disorder	Gastrointestinal Disorder/Crohn’s Disease	Overall Medicinal Cannabis Cohort
	OG Shark (43; 10.5%)	Sweet Skunk CBD (13; 8.8%)	Jack Herer (52; 10.8%)	Lemon Sour Diesel (20; 13.8%)	Island Sweet Skunk (8; 9.8%)	OG Shark (120; 8.6%)
	CBD House Blend (34; 8.3%)	OG Shark (12; 8.1%)	Island Sweet Skunk (50; 10.4%)	OG shark (15; 10.4%)	Jack Herer (8; 9.8%)	Jack Herer (119; 8.5%)
	Pink Kush (34; 8.3%)	Cannatonic (11; 7.4%)	White Widow (46; 9.6%)	Skywalker OG (13; 9%)	Black Tuna (7; 8.5%)	White Widow (109; 7.8%)
	Skywalker OG (29; 7.1%)	CBD House Blend (10; 6.8%)	Jean Guy (41; 8.5%)	Pink Kush (12; 8.3%)	Afghani (6; 7.3%)	Lemon Sour Diesel (109; 7.8%)
	Master Kush (28; 6.8%)	Indica House Blend (9; 6.1%)	Lemon Sour Diesel (37; 7.7%)	Jack Herer (10; 6.9%)	Warlock CBD (6; 7.3%)	Pink Kush (109; 7.8%)
	Warlock CBD (28; 6.8%)	Jack Herer (9; 6.1%)	Pink Kush (35; 7.3%)	White Widow (9; 6.2%)	White Widow (6; 7.3%)	Island Sweet Skunk (107; 7.6%)
	Black Tuna (27; 6.6%)	Warlock CBD (8; 5.4%)	OG Shark (34; 7.1%)	Afghani (8; 5.5%)	CBD House Blend (5; 6.1%)	Jean Guy (95; 6.8%)
	Jean Guy (26; 6.3%)	Lemon Sour Diesel (8; 5.4%)	Sweet Skunk CBD (30; 6.2%)	Indica House Blend (7; 4.8%)	Sweet Skunk CBD (5; 6.1%)	Skywalker OG (90; 6.4%)
	Lemon Sour Diesel (26; 6.3%)	White Widow (8; 5.4%)	Afghani (28; 5.8%)	Sweet Skunk CBD (7; 4.8%)	Hybrid House Blend (5; 6.1%)	Afghani (87; 6.2%)
	Jack Herer (25; 6.1%)	Island Sweet Skunk (8; 5.4%)	Skywalker OG (24; 5%)	Island Sweet Skunk (7; 4.8%)	Pink Kush (5; 6.1%)	Sweet Skunk CBD (81; 5.8%)
	Cannatonic (24; 5.8%)	Hybrid House Blend (7; 4.7%)	Master Kush (24; 5%)	Black Tuna (7; 4.8%)	Cannatonic (4; 4.9%)	Cannatonic (77; 5.5%)
	White Widow (24; 5.8%)	Master Kush (7; 4.7%)	Hybrid House Blend (23; 4.8%)	Jean Guy (6; 4.1%)	Lemon Sour Diesel (4; 4.9%)	Warlock CBD (77; 5.5%)
	Island Sweet Skunk (22; 5.4%)	Pink Kush (7; 4.7%)	Warlock CBD (21; 4.4%)	Rock Star (6; 4.1%)	Headband (4; 4.9%)	CBD House Blend (76; 5.4%)
	Sweet Skunk CBD (21; 5.1%)	Skywalker OG (7; 4.7%)	Cannatonic (20; 4.2%)	Sour Diesel (6; 4.1%)	OG Shark (3; 3.7%)	Master Kush (75; 5.4%)
	Headband (20; 4.9%)	Afghani (6; 4.1%), Blueberry (6; 4.1%), Girl Scout Cookies (GSC) (6; 4.1%), Jean Guy (6; 4.1%)	Black Tuna (16; 3.3%)	Master Kush (6; 4.1%), Mango (6; 4.1%)	Jean Guy (3; 3.7%), Blueberry (3; 3.7%), Purple Kush (3; 3.7%)	Black Tuna (70; 5%)

**Table 6:** Preferred medicinal cannabis types and strains in all non-headache groups, excluding patients with headache as primary symptom

Preferred Cannabis Type						
	Chronic pain as primary illness (450)	Arthritis as primary illness (159)	Mental Health Condition (417)/PTSD (69) = (486)	Insomnia/Sleep Disorder (166)	Gastrointestinal Disorder (51)/Crohn’s Disease (32) = (83)	Overall Medicinal Cannabis Cohort (1527)
Hybrid	162 (36%)	46 (28.9%)	138 (28.4%)	52 (31.3%)	33 (39.8%)	486 (31.8%)
Indica	114 (25.3%)	51 (32.1%)	125 (25.7%)	74 (44.6%)	10 (12.1%)	426 (27.9%)
Sativa	88 (19.6%)	26 (16.4%)	154 (31.7%)	32 (19.3%)	20 (24.1%)	366 (24%)
3:1 CBD:THC	40 (8.9%)	17 (10.7%)	37 (7.6%)	2 (1.2%)	10 (12.1%)	120 (7.9%)
1:1 CBD:THC	43 (9.6%)	16 (10.1%)	30 (6.2%)	6 (3.6%)	10 (12.1%)	121 (7.9%)
No response	3 (0.7%)	3 (1.9%)	2 (0.4%)	0 (0%)	0 (0%)	8 (0.5%)
Preferred Cannabis Strains (top 15)						
	Chronic pain as primary illness	Arthritis as primary illness	Mental Health Condition + PTSD	Insomnia/Sleep Disorder	Gastrointestinal Disorder + Crohn’s Disease	Overall Medicinal Cannabis Cohort
	OG Shark (33; 10.5%)	OG Shark (11; 9.3%)	Jack Herer (42; 11.6%)	Lemon Sour Diesel (17; 14.4%)	Island Sweet Skunk (7; 10.6%)	Jack Herer (93; 8.9%)
	Pink Kush (30; 9.6%)	Cannatonic (10; 8.5%)	Island Sweet Skunk (39; 10.7%)	OG shark (10; 8.5%)	Jack Herer (6; 9%)	Pink Kush (87; 8.3%)
	CBD House Blend (29; 9.3%)	Sweet Skunk CBD (9; 7.6%)	White Widow (38; 10.5%)	Skywalker OG (10; 8.5%)	Warlock CBD (6; 9%)	OG Shark (86; 8.2%)
	Skywalker OG (22; 7%)	CBD House Blend (9; 7.6%)	Jean Guy (28; 7.7%)	Pink Kush (10; 8.5%)	Sweet Skunk CBD (5; 7.6%)	Island Sweet Skunk (86; 8.2%)
	Warlock CBD (21; 6.7%)	Jack Herer (9; 7.6%)	Pink Kush (27; 7.4%)	Jack Herer (9; 7.6%)	White Widow (5; 7.6%)	White Widow (83; 7.9%)
	Jack Herer (20; 6.4%)	Indica House Blend (8; 6.8%)	Lemon Sour Diesel (26; 7.2%)	White Widow (9; 7.6%)	Hybrid House Blend (5; 7.6%)	Lemon Sour Diesel (81; 7.7%)
	Master Kush (19; 6.1%)	Warlock CBD (7; 5.9%)	OG Shark (23; 6.3%)	Afghani (7; 5.9%)	Afghani (4; 6%)	Jean Guy (65; 6.2%)
	Black Tuna (19; 6.1%)	Lemon Sour Diesel (7; 5.9%)	Sweet Skunk CBD (21; 5.8%)	Black Tuna (7; 5.9%)	Black Tuna (4; 6%)	Warlock CBD (63; 6%)
	Afghani (18; 5.8%)	White Widow (7; 5.9%)	Afghani (20; 5.5%)	Sweet Skunk CBD (6; 5.1%)	Lemon Sour Diesel (4; 6%)	CBD House Blend (63; 6%)
	Lemon Sour Diesel (18; 5.8%)	Pink Kush (7; 5.9%)	Warlock CBD (20; 5.5%)	Island Sweet Skunk (6; 5.1%)	Headband (4; 6%)	Skywalker OG (62; 5.9%)
	Island Sweet Skunk (18; 5.8%)	Hybrid House Blend (6; 5.1%)	Cannatonic (18; 5%)	Indica House Blend (6; 5.1%)	Cannatonic (4; 6%)	Sweet Skunk CBD (60; 5.7%)
	Sweet Skunk CBD (17; 5.4%)	Master Kush (6; 5.1%)	Master Kush (17; 4.7%)	Master Kush (6; 5.1%)	CBD House Blend (3; 4.6%)	Afghani (59; 5.6%)
	Cannatonic (17; 5.4%)	Island Sweet Skunk (6; 5.1%)	Skywalker OG (16; 4.4%)	Jean Guy (5; 4.2%)	Purple Kush (3; 4.6%)	Cannatonic (59; 5.6%)
	Jean Guy (17; 5.4%)	Girl Scout Cookies (GSC) (6; 5.1%)	Hybrid House Blend (15; 4.1%)	Blueberry (5; 4.2%)	Jean Guy (3; 4.6%)	Master Kush (54; 5.1%)
	Girl Scout Cookies (GSC) (15; 4.8%)	Skywalker OG (5; 4.2%), Jean Guy (5; 4.2%)	Black Tuna (13; 3.6%)	Mango (5; 4.2%)	Pink Kush (3; 4.6%)	Black Tuna (50; 4.8%)

Statistical analysis was performed to determine if there were significant differences in preferred cannabis types reported by headache patients. The data were insufficient for statistical analysis of specific strain preferences. There were no statistically significant differences found between patients with headache as primary illness and those with chronic pain, arthritis, or mental health condition/PTSD. When compared to insomnia/sleep disorder patients, headache as primary illness patients were 7.7 times as likely to prefer 3:1 CBD:THC over Indica (OR 7.7, 95% CI 1.7-35.11, *p* = .003).

Patients with headache as primary symptom were 2.7 times as likely to prefer Sativa over 1:1 CBD:THC (OR 2.66, 95% CI 1.52-4.66, *p* < .001) when compared to chronic pain patients. When compared to arthritis patients, headache as primary symptom patients were 3.4 times as likely to prefer Sativa over 1:1: CBD:THC (OR 3.35, 95% CI 1.57-7.12, *p* = .001). When compared to insomnia patients, headache as primary symptom patients were over twice as likely to prefer Sativa over Indica (OR 2.18, 95% CI 1.36-3.52, *p* = .001) and 8.7 times as likely to prefer 3:1 CBD:THC over Indica (OR 8.74, 95% CI 2.04-37.37, *p* < .001). When compared to gastrointestinal disorder/Crohn’s disease patients, headache as primary symptom patients were almost three times as likely to prefer Indica over Hybrid (OR 2.88, 95% CI 1.37-6.05, *p* = .004), 4.2 times as likely to prefer Indica over 3:1 CBD:THC (OR 4.24, 95% CI 1.63-10.98, *p* = .002), and 5.8 times as likely to prefer Indica over 1:1 THC:CBD (OR 5.76, 95% CI 2.17-15.26, *p* < .001). There were no statistically significant differences found between headache as primary symptom patients and mental health condition/PTSD patients, nor between all non-headache patients as a group.

A number of variables were assessed across all pain groups. These variables included primary method of cannabis use, prevalence of cannabis extract (drops, capsules) use and preferences, cannabis quantity and frequency of use, highest level of education completed, employment status, and prescription medications replaced with medicinal cannabis. The most common primary methods of use across all pain groups were vaporizing and joint use, although additional methods included waterpipe/bong, oral (edibles such as oil drops/extracts, baked goods, butter, tincture), pipe, juicing, tea, or topical use, as seen in Table 7. In the 505 patients with headache as a primary symptom, the most common primary methods of use were joint in 170 (33.7%), and vaporizing in 162 (32.1%), and this pattern was similar in the positive ID Migraine™ groups. In general, primary methods of use were similar to the top non-pain related primary illnesses, and the overall patient cohort.

**Table 7:** Primary method of medicinal cannabis use among various pain syndromes, “Yes” responses on ID Migraine™ questionnaire, top non-pain related primary illnesses, and overall cohort

Primary method of use								
	Vaporizer	Pipe	Joint	Oral/ Edible	Waterpipe/ Bong	Juicing	Tea	Topical
Headache as primary symptom (505)	162 (32.1%)	50 (9.9%)	170 (33.7%)	58 (11.5%)	63 (12.5%)	1 (0.2%)	1 (0.2%)	
Headache as primary illness (75)	26 (34.7%)	8 (10.7%)	22 (29.3%)	9 (12%)	8 (10.7%)	1 (1.3%)	1 (1.3%)	
Chronic pain as primary illness (598)	179 (29.9%)	56 (9.4%)	183 (30.6%)	120 (20.1%)	56 (9.4%)	1 (0.17%)		3 (0.5%)
Arthritis as primary illness (188)	70 (37.2%)	16 (8.5%)	60 (31.9%)	36 (19.2%)	4 (2.1%)			2 (1.1%)
3 Yes (343)^a^	109 (31.8%)	37 (10.8%)	120 (35%)	37 (10.8%)	39 (11.4%)		1 (0.29%)	
2 Yes (102)^b^	34 (33.3%)	9 (8.8%)	29 (28.4%)	11 (10.8%)	19 (18.6%)			
3 + 2 Yes (445)	143 (32.1%)	46 (10.3%)	149 (33.5%)	48 (10.8%)	58 (13%)			
Mental Health Condition (548) + PTSD (93)	184 (28.7%)	89 (13.9%)	195 (30.4%)	74 (11.5%)	97 (15.1%)	1 (0.16%)	1 (0.16%)	
Insomnia/Sleep Disorder (198)	63 (31.8%)	19 (9.6%)	65 (32.8%)	30 (15.2%)	19 (9.6%)	1 (0.51%)		1 (0.51%)
Gastrointestinal Disorder (62) + Crohn’s Disease (35)	34 (35.1%)	12 (12.4%)	26 (26.8%)	11 (11.3%)	14 (14.4%)			
Overall Medicinal Cannabis Cohort (2032)	632 (31.1%)	229 (11.3%)	617 (30.4%)	330 (16.2%)	212 (10.4%)	4 (0.20%)	2 (0.10%)	6 (0.30%)

^a^3 “Yes” responses = 97% probability of migraine

^b^2 “Yes” responses = 93% probability of migraine

The majority of patients using cannabis extracts (drops, capsules) across pain groups preferred the 3:1 CBD:THC extract with the exception that the chronic pain group preferred 1:1 CBD:THC extract, the 3 “Yes” positive ID Migraine™ group preferred Indica extract, and the combined 3 + 2 “Yes” positive ID Migraine™ group equally preferred 3:1 CBD:THC and Indica extracts, as seen in Table 8. Overall, in the headache as primary symptom group, 195 (38.6%) were using cannabis extracts, and the 3:1 CBD:THC extract was most commonly used in 53 (27.2%) followed by the Indica extract in 51 (26.2%).

**Table 8:** Medicinal cannabis extract use preferences among various pain syndromes and “Yes” responses on ID Migraine™ questionnaire

Cannabis extracts (drops, capsules)						
	Total	Hybrid	Indica	Sativa	3:1 CBD:THC	1:1 CBD:THC
Headache as primary symptom (505)	195 (38.6%)	36 (18.5%)	51 (26.2%)	15 (7.7%)	53 (27.2%)	40 (20.5%)
Headache as primary illness (75)	26 (34.7%)	7 (26.9%)	5 (19.2%)	1 (3.9%)	9 (34.6%)	4 (15.4%)
Chronic pain as primary illness (598)	248 (41.5%)	44 (17.7%)	56 (22.6%)	18 (7.3%)	60 (24.2%)	66 (26.6%)
Arthritis as primary illness (188)	80 (42.6%)	14 (17.5%)	11 (13.8%)	5 (6.3%)	26 (32.5%)	24 (30%)
3 Yes (343)^a^	143 (41.7%)	25 (17.5%)	41 (28.7%)	6 (4.2%)	39 (27.3%)	32 (22.4%)
2 Yes (102)^b^	33 (32.4%)	6 (18.2%)	7 (21.2%)	5 (15.2%)	9 (27.3%)	6 (18.2%)
3 + 2 Yes (445)	176 (39.6%)	31 (17.6%)	48 (27.3%)	11 (6.3%)	48 (27.3%)	38 (21.6%)

^a^3 “Yes” responses = 97% probability of migraine

^b^2 “Yes” responses = 93% probability of migraine

Quantity of cannabis used was estimated as one joint = 0.3-0.5 g, one eighth = 3.5 g, one quarter = 7 g, and one ounce = 28 g. The quantity and frequency of medicinal cannabis use across the groups ranged from 9.6-11.4 g/week, 1.4-1.7 g/day, 0.58-0.76 g/treatment, 5.9-6.5 days/week and 3.2-3.9 times/day. The quantity of medicinal cannabis use in the headache group averaged 11.4 g/week, 1.7 g/day, and 0.66 g/treatment, with a frequency of 6.4 days/week, and 3.9 times/day. The positive ID Migraine™ patients averaged similar patterns of use, although at the upper ranges of use. These results can all be seen in Table 9.

**Table 9:** Quantity and frequency of medicinal cannabis use among various pain syndromes and “Yes” responses on ID Migraine™ questionnaire

Cannabis quantity and frequency used					
	Grams per week (Average)	Grams per day (Average)	Grams per treatment (Average)	Days used per week (Average)	Times used per day (Average)
Headache as primary symptom (505)	1 to > 28 (11.4)	≤0.25 to ≥4 (1.7)	≤0.25 to ≥4 (0.66)	1-7 (6.4)	1 to > 10 (3.9)
Headache as primary illness (75)	1 to > 28 (9.6)	≤0.25 to ≥4 (1.4)	≤0.25 to ≥4 (0.67)	1-7 (5.9)	1 to > 10 (3.3)
Chronic pain as primary illness (598)	1 to > 28 (10.8)	≤0.25 to ≥4 (1.6)	≤0.25 to ≥4 (0.68)	1-7 (6.2)	1 to > 10 (3.7)
Arthritis as primary illness (188)	1 to > 28 (9.8)	≤0.25 to ≥4 (1.4)	≤0.25 to ≥4 (0.58)	1-7 (6.1)	1 to > 10 (3.2)
3 Yes (343)^a^	1 to > 28 (11.2)	≤0.25 to ≥4 (1.7)	≤0.25 to ≥4 (0.63)	1-7 (6.4)	1 to > 10 (3.9)
2 Yes (102)^b^	1 to > 28 (11.3)	≤0.25 to ≥4 (1.7)	≤0.25 to ≥4 (0.76)	1-7 (6.5)	1 to > 10 (3.8)
3 + 2 Yes (445)	1 to > 28 (11.3)	≤0.25 to ≥4 (1.7)	≤0.25 to ≥4 (0.70)	1-7 (6.5)	1 to > 10 (3.9)

^a^3 “Yes” responses = 97% probability of migraine

^b^2 “Yes” responses = 93% probability of migraine

The highest level of education completed across medicinal cannabis user groups can be seen in Table 10. Options included graduate degree, university degree (Bachelors’ degree or equivalent), some college/university but no degree/certificate, technical/non-university degree, high school degree or equivalent (GED), and less than high school degree. The most common education level completed across all pain groups was technical/non-university degree, including the headache group, *n* = 158 (31.3%). The exception was in the 2 “Yes” positive ID Migraine™ group, which most commonly reported some college/university but no degree/certificate.

**Table 10:** Highest education level completed and employment status in medicinal cannabis users among various pain syndromes and “Yes” responses on ID Migraine™ questionnaire

Highest level of education completed						
	Graduate degree	University degree (Bachelors’ degree or equivalent)	Some college/university, but no degree/certificate	Technical and non-university degree	High school degree or equivalent (GED)	Less than high school degree
All patients (2032)	122 (6%)	322 (15.9%)	432 (21.3%)	642 (31.6%)	375 (18.5%)	139 (6.8%)
Headache as primary symptom (505)	17 (3.4%)	81 (16%)	124 (24.6%)	158 (31.3%)	91 (18%)	34 (6.7%)
Headache as primary illness (75)	5 (6.7%)	18 (24%)	16 (21.3%)	22 (29.3%)	9 (12%)	5 (6.7%)
Chronic pain as primary illness (598)	39 (6.5%)	74 (12.4%)	131 (21.9%)	196 (32.8%)	107 (17.9%)	51 (8.5%)
Arthritis as primary illness (188)	10 (5.3%)	31 (16.5%)	36 (19.2%)	65 (34.6%)	38 (20.2%)	8 (4.3%)
3 Yes (343)^a^	10 (2.9%)	54 (15.7%)	87 (25.4%)	114 (33.2%)	53 (15.5%)	25 (7.3%)
2 Yes (102)^b^	4 (3.9%)	13 (12.8%)	30 (29.4%)	28 (27.5%)	21 (20.6%)	6 (5.9%)
3 + 2 Yes (445)	14 (3.2%)	67 (15.1%)	117 (26.3%)	142 (31.9%)	74 (16.6%)	31 (7.0%)
Employment status						
	Employed, working full-time	Employed, working part-time	Retired	Not employed, looking for work	Not employed, not looking for work	Disabled, not able to work
All patients (2032)	1045 (51.4%)	231 (11.4%)	120 (5.9%)	164 (8.1%)	88 (4.3%)	384 (18.9%)
Headache as primary symptom (505)	268 (53.1%)	50 (9.9%)	10 (2%)	36 (7.1%)	30 (5.9%)	111 (22%)
Headache as primary illness (75)	56 (74.7%)	4 (5.3%)	1 (1.3%)	1 (1.3%)	5 (6.7%)	8 (10.7%)
Chronic pain as primary illness (598)	278 (46.5%)	64 (10.7%)	33 (5.5%)	30 (5%)	24 (4%)	169 (28.3%)
Arthritis as primary illness (188)	94 (50%)	18 (9.6%)	38 (20.2%)	13 (6.9%)	4 (2.1%)	21 (11.2%)
3 Yes (343)^a^	172 (50.2%)	31 (9%)	6 (1.8%)	24 (7%)	21 (6.1%)	89 (26%)
2 Yes (102)^b^	59 (57.8%)	12 (11.8%)	2 (2%)	9 (8.8%)	3 (2.9%)	17 (16.7%)
3 + 2 Yes (445)	231 (51.9%)	43 (9.7%)	8 (1.8%)	33 (7.4%)	24 (5.4%)	106 (23.8%)

^a^3 “Yes” responses = 97% probability of migraine

^b^2 “Yes” responses = 93% probability of migraine

Employment status among medicinal cannabis users was assessed, and can be seen in Table 10. The options were employed working full-time, employed working part-time, retired, not employed looking for work, not employed not looking for work, and disabled not able to work. The vast majority of patients across all pain groups were employed working full time, including the headache group, *n* = 268 (53.1%).

Prescription medications that were replaced with medicinal cannabis were also recorded, as seen in Table 11, and included opiates/opioids, NSAIDs/analgesics, triptans, ergots, anti-depressant/anti-anxiety, anti-convulsant, and muscle relaxers. Many patients across all groups had replaced prescription medications with medicinal cannabis, including headache as primary symptom *n* = 272 (53.9%). Ranges of prescription medication replacement across pain groups varied between 41.2%-59.5% of patients. The most common prescription medications replaced by medicinal cannabis were opiates/opioids in every pain group, including headache as primary symptom *n* = 118 (43.4%). Ranges of opiate/opioid replacement across pain groups varied between 40.5%-72.8% of patients. Notably, additional prescription medications replaced by medicinal cannabis in headache patients included 106 (39%) anti-depressant/anti-anxiety, 57 (21%) NSAIDs, 22 (8.1%) triptans, 21 (7.7%) anticonvulsants, 19 (7%) muscle relaxers, and 1 (0.4%) ergots.

**Table 11:** Medicinal cannabis reported as a substitute for prescription drugs among various pain syndromes and “Yes” responses on ID Migraine™ questionnaire

Prescription drugs replaced							
	Yes	Opiates, opioids	NSAIDs, Analgesics	Triptans/Ergots	Anti-depressant, Anti-anxiety	Anti-convulsant	Muscle Relaxers
Headache as primary symptom (505)	272 (53.9%)	118 (43.4%)	57 (21%)	22 (8.1%)/1 (0.4%)	106 (39%)	21 (7.7%)	19 (7%)
Headache as primary illness (75)	36 (48%)	19 (52.8%)	11 (30.6%)	14 (38.9%)	5 (13.9%)	1 (2.8%)	4 (11.1%)
Chronic pain as primary illness (598)	316 (52.8%)	230 (72.8%)	64 (20.3%)	3 (1%)	74 (23.4%)	41 (13%)	30 (9.5%)
Arthritis as primary illness (188)	90 (47.9%)	48 (53.3%)	37 (41.1%)	2 (2.2%)	15 (16.7%)	5 (5.6%)	7 (7.8%)
3 Yes (343)^a^	204 (59.5%)	92 (45.1%)	45 (22.1%)	20 (9.8%)/1 (0.5%)	84 (41%)	13 (6%)	15 (7.4%)
2 Yes (102)^b^	42 (41.2%)	17 (40.5%)	6 (14.3%)	2 (4.8%)	15 (35.7%)	6 (14.3%)	4 (9.5%)
3 + 2 Yes (445)	246 (55.3%)	109 (44.3%)	51 (20.7%)	22 (8.9%)/1 (0.4%)	99 (40.2%)	19 (7.7%)	19 (7.7%)

^a^3 “Yes” responses = 97% probability of migraine

^b^2 “Yes” responses = 93% probability of migraine

## Discussion

The neurobiological pathways of cannabinoids and pain, including migraine and headache, have been detailed, summarized and should be reviewed [1, 2, 51, 65, 68–70]. Briefly, the endocannabinoid system is distributed throughout the central and peripheral nervous system, is involved in inflammatory and pain processing, and plays regulatory physiological roles across virtually every organ system [19, 46, 71–74]. The endocannabinoid system interacts within its own pathways, as well as within major endogenous pain pathways, including inflammatory, endorphin/enkephalin, vanilloid/transient receptor potential cation channel subfamily V (TRPV), subfamily M (TRPM), subfamily A (TRPA), and nuclear receptors/transcription factors called the peroxisome proliferator-activated receptors (PPAR) [75].

The activities of the endocannabinoid system are based on the pre-synaptic G protein-coupled cannabinoid 1 (CB1) and 2 (CB2) receptors [76]. There is also a presumed third cannabinoid receptor, G protein-coupled receptor 55 (GPR55), termed CB3 [77]. The primary endogenous cannabinoid receptor ligands (endogenous cannabinoids, or endocannabinoids) are arachidonic acid derivatives, and they work via retrograde signaling receptor activation. The primary mediator of endocannabinoid signaling is N-arachidonoylethanolamine (anandamide, or AEA), and 2-arachidonoylglycerol (2-AG) is another primary endocannabinoid [71, 78–80]. Cannabis-based phytocannabinoids, as well as inherent endocannabinoids interact at the CB1 and CB2 receptors with variable affinities and actions [81–83].

The CB1 receptor is the most abundant G protein-coupled receptor in the brain and one of the most abundant in both the peripheral and central nervous system [81]. CB1 receptors are expressed primarily on presynaptic peripheral and central nerve terminals, and are found extensively through the anatomical pain pathways as well as many other neurological central and peripheral locations [19, 84–87]. CB1 receptors are associated with the “high” felt with some cannabis strains, activated by THC. Activation leads to hyperpolarization of the pre-synaptic terminal, closing of calcium channels with subsequent inhibition of released stored inhibitory and excitatory neurotransmitters, including glutamate, 5-hydroxytryptamine (5-HT; serotonin), gamma-aminobutyric acid (GABA), noradrenaline, dopamine, acetylcholine, D-aspartate, and cholecystokinin at inhibitory and excitatory synapses [19, 71, 73, 80, 86, 88–90], and can modulate pain pathways involving opioid, serotonin, and N-methyl-d-aspartate (NMDA) receptors through other indirect mechanisms [91].

The CB2 receptors are located primarily in the peripheral tissues and immune cells where they influence the release of cytokines, chemokines, and cell migration including neutrophils and macrophages, but do have some presence in the central nervous system [18, 86, 92–95], and may also contribute to pain relief by dopamine release modulation [96, 97].

Over 540 phytochemicals have been described in cannabis [98], 18 different chemical classes, and more than 100 different phytocannabinoids, although some are breakdown products [99, 100]. THC and CBD have been the most researched and are considered the major cannabinoids. There are many additional cannabinoids referred to as minor cannabinoids. The quantities of major and minor cannabinoids are widely variable between different types of cannabis strains. There is evidence for analgesic and anti-inflammatory effects in many of the cannabinoids, and this publication will focus primarily on these properties for the cannabinoids assessed in this study. However, a more extensive discussion and a comprehensive review of other medicinal properties of these, as well as many other cannabinoids, has been summarized and is available [28]. The cannabinoids analyzed in this study were limited to THC, THCA, CBD, and CBDA.

THC is one of the most researched cannabinoids, and the cause of the psychoactive side effects of cannabis, suspected from modulation of glutamate and GABA systems [18, 83, 101–103]. It is a partial agonist at CB1 greater than CB2 receptors, which are its primary mechanisms of action. However, other mechanisms of action reflect its activity as an agonist at the PPAR-γ and TRPA1 receptors [83], a 5HT3A antagonist, a glycine receptor activation enhancer via allosteric modification, reduces elevated intracellular calcium levels from TRPM8 activity (cold and menthol receptor 1 (CMR1)), elevates calcium levels by TRPA1 or TRPV2, and stimulates G Protein Receptor 18 and other nuclear receptors [104–113]. It reduces NMDA responses by 30-40% [114–116], blocks capsaicin-induced hyperalgesia [117], inhibits CGRP activity [118], increases cerebral 5HT production, decreases 5HT reuptake, and inhibits 5HT release from platelets, all of which may influence trigeminovascular migraine circuitry [1, 68, 69, 119]. THC enhances analgesia from kappa opioid receptor agonist medications [120–123], stimulates production of beta-endorphin and increases proenkephalin mRNA levels in brainstem regions involved in pain processing [124–126], and intraventricular and intrathecal administration of THC produces analgesia similar to opioids [127].

THC is 20 times more anti-inflammatory than aspirin, twice as anti-inflammatory as hydrocortisone [128], and has well documented analgesic and anti-inflammatory benefits including arthritic and inflammatory conditions [83, 114, 127, 129–156]. There have been many positive studies across various chronic pain syndromes, showing benefit of THC in trials with smoked or vaporized cannabis comparing between different doses of THC, with benefit often noted at higher percentages [28, 47, 157–169]. However, compositions of other cannabinoids including CBD, minor cannabinoids, and other important compounds such as terpenes were not assessed in most of these trials. Given the entourage effects of cannabis [100, 170], where cannabinoids and terpenes influence activity of one another, resulting in strain-specific characteristics, effects and responses, it is often unclear if these studies showing positive (or negative) effects of cannabis are due to the THC alone, or due to synergy between undefined compositions of other cannabinoids and terpenes.

There have been a multitude of studies confirming benefit in various chronic pain syndromes with an oral-mucosal spray called Nabiximols (Sativex) [171–196], approved in 30 countries for various neurological symptoms. This is a tincture of cannabis made from cannabis plants [197]. Each spray delivers a standardized dose of 2.7 mg THC and 2.5 mg CBD, along with additional cannabinoids, flavonoids, and terpenes in unmeasured small amounts. Despite the standardized THC:CBD ratio, the actual concentrations of terpenes and other compounds are unknown. This again creates uncertainty as to what components are providing most of the benefit, although entourage effects are again suspected. There was also a study comparing between three varieties of this spray; 1:1 THC:CBD vs. THC alone vs. CBD alone and the sprays that contained THC showed the most pain benefit, over CBD alone [179]. Other cannabis extract studies of only THC and CBD in varying doses also showed pain benefit, although these did not evaluate each cannabinoid individually [187, 198].

The strong anti-emetic benefits of THC have also been well documented in adults [26, 83, 129, 130, 199–238] and children [235, 239–241], and migraine associated nausea and vomiting would certainly be another benefit of THC. In fact, the FDA has approved two synthetic forms of THC in the treatment of chemotherapy related nausea and vomiting; Dronabinol [242] and Nabilone [243]. Notably, these synthetic THC medications have also shown analgesic effects [55, 57, 62, 188, 244–256].

Besides THC, CBD is the other major cannabinoid. It has gained a lot of attention over the past several years due to its lack of any psychoactivity, as opposed to THC. In November 2017, The World Health Organization announced that in humans, CBD exhibits no evidence for abuse or dependence potential, and there is no evidence of public health related problems associated with the use of pure CBD [257]. In January 2018, the World Anti-Doping Agency (WADA) removed CBD from their prohibited list, no longer banning use by athletes [258]. CBD has powerful analgesic and anti-inflammatory effects [23, 83, 114, 129–131, 137–140, 149, 259–281] mediated by both cyclooxygenase and lipoxygenase inhibition. Its anti-inflammatory effect is several hundred times more potent than aspirin [128, 282], although to date, there have been no clinical studies evaluating pure CBD in headache or chronic pain disorders. CBD has much lower affinity for CB1 or CB2 receptors, and acts as an antagonist of CB1 and CB2 agonists such as THC [276]. At low concentrations, its antagonism of CB1 underlies its neutralizing effects on the CB1 agonist THC side effects such as anxiety, tachycardia, and sedation [283–288]. CBD appears to attenuate some of these negative side effects of THC when the CBD:THC ratio is at least 8:1 (± 11.1), but may potentiate some of the THC side effects when the CBD:THC ratio is around 2:1 (± 1.4) [286, 288]. It is also an inverse agonist at the CB2 receptor, which may contribute to its anti-inflammatory effects [276].

CBD also interacts with a multitude of ion channels, enzymes, and other receptors [18, 83, 129, 130, 225, 259]. It acts as a TRPV1 agonist, similar to capsaicin, although without the noxious sides effects, and also inhibits AEA uptake and metabolism [108–110, 289, 290]. It acts as a positive allosteric modulator at α1 and α1β glycine receptors [291], suggested to play a role in chronic pain after inflammation or nerve injury since glycine acts as an inhibitory postsynaptic neurotransmitter in the dorsal horn of the spinal cord. CBD acts as a μ opioid receptor ligand and a positive allosteric modulator at μ and δ opioid receptors suggesting that it may enhance opiate effects [83]. Additional mechanisms of action suggested to reflect its anti-inflammatory and analgesic effects, as well as other medicinal benefits, include TRPA1 agonist, TRPV1 agonist, TRPM8 antagonist [108–110], TRPV2 agonist in which it may mediate CGRP release from dorsal root ganglion neurons [292], T-type calcium^2+^ channel inhibitor [293], suppression of tryptophan degradation (precursor to 5HT) [294], phospholipase A2 modulator [295], 5-HT1A agonist [83, 296], regulator of intracellular calcium^2+^ [297, 298], fatty acid amide hydrolase (FAAH; breaks down AEA) inhibition [290], GPR55 antagonist [77], adenosine uptake competitive inhibitor [299], PPARγ agonist [300], 5-lipoxygenase and 15-lipoxygenase inhibitors [301], and antagonism of the abnormal-CBD receptor [83, 302].

Cannabinoid acids are the precursors to the cannabinoids in raw and live cannabis, and have no psychotropic qualities. They are decarboxylated by heat, UV exposure, and prolonged storage to form the active cannabinoids, although heat such as from smoking or vaporizing is the primary conversion factor. The two cannabinoid acids assessed in this study were THCA, which converts to THC, and CBDA, which converts to CBD.

THCA is a TRPA1 partial agonist [108], and TRPM8 antagonist [108] which may underlie a potential role in analgesia, and has been shown to have anti-inflammatory [140] and anti-nausea properties [303]. CBDA is often obtained through consumption of raw cannabis juice. It is a TRPA1 agonist [108], TRPV1 agonist [290], and TRPM8 antagonist [108] which may also reflect its potential as an analgesic. It is also anti-inflammatory [130, 140, 304] via selective COX2 inhibition, and has anti-nausea properties [237, 305].

The terpenes, or terpenoids, form the largest group of phytochemicals [99], and account for some pharmacological properties of cannabis, as well as many medicinal herbs, plants and essential oils. They are the source of flavors, aromas, and other characteristics that help differentiate cannabis strains. The terms terpenes and terpenoids are often used interchangeably in the literature, although technically, terpenes are basic hydrocarbons, while terpenoids contain extra functional groups of a wide range of chemical elements. Cannabis contains up to 200 different terpenes [100], and they are generally classified as primary and secondary terpenes, based on how frequent they occur in cannabis. They are lipophilic with wide ranging mechanisms of action sites including neurotransmitter receptors, G-protein receptors, muscle and neuronal ion channels, enzymes, cell membranes, and second messenger systems [100, 306, 307]. The terpenes work synergistically with the cannabinoids for a variety of therapeutic effects, and this phenomenon is known as the cannabis entourage effects [100, 170]. They have shown many medicinal benefits, including anti-inflammatory and analgesic properties [308]. This publication will focus primarily on the anti-inflammatory and analgesic evidence for the terpenes analyzed in this study, although a more extensive discussion and a comprehensive review of other medicinal properties of these, as well as many other terpenes has been summarized and is available [28]. The majority of this data comes from preclinical studies involving animal models or in vitro studies, and some of the reported benefits attributed to individual terpenes come from studies evaluating whole essential oils or plants in which the specified terpene may be a predominant constituent. However, therapeutic contribution from some of the other terpenes in some of these studies cannot be excluded. The terpenes analyzed in this study were limited to α-pinene, β-myrcene, D-limonene, linalool, β-caryophyllene, humulene, trans-nerolidol, and bisabolol.

Alpha-pinene (α-pinene) is the most commonly occurring terpene in nature [309], and accounts for the aroma of fresh sage, pine needles, and conifers, but is produced by many herbs such as basil, parsley, and dill as well. It has anti-inflammatory effects in human chondrocytes, suggesting anti-osteoarthritic activity [310, 311], anti-inflammatory effects by PGE-1 [312], and anti-nociception properties [313].

Beta-myrcene (β-myrcene), or myrcene, is common in lemongrass, basil, bay leaves, wild thyme, parsley, hops, and tropical fruits such as mango. It has potent anti-inflammatory, analgesic, and anxiolytic properties [314–316], and has benefit in muscle relaxation [317], and prominent sedation/hypnotic, helpful in sleep [317, 318]. Its analgesic effects were antagonized by naloxone suggesting an opioid-mediated mechanism [315, 316]. Its significant anti-inflammatory effects [319] occur via prostaglandin E2 [315] and it has anti-catabolic effects in human chondrocytes suggesting anti-osteoarthritic activity and the ability to halt or slow down cartilage destruction and osteoarthritis progression [320].

D-limonene (limonene) is prominent in the rinds of citrus fruits, and the second most commonly occurring terpene in nature [309]. It has analgesic [321], anti-inflammatory [320, 322–325], and antidepressant effects [321, 326]. It contributes to muscle relaxation and sleep [317], and is a powerful anxiolytic [327–330], which extended anxiolytic benefit to patients with chronic myeloid leukemia (CML) [331]. It increases the metabolic turnover of dopamine in the hippocampus and serotonin in the prefrontal cortex and striatum, suggesting that anxiolytic and antidepressant-like effects may occur by the suppression of dopamine activity related to enhanced serotonergic neurons, especially via 5-HT1A [332].

Linalool is found in flowers and spices including citrus, lavender, rosewood, birch trees, and coriander. It exhibits anti-inflammatory and analgesic activity [333–335] as well as anti-nociception via activation of opioidergic and cholinergic systems [333], anticonvulsant via anti-glutamatergic and GABA neurotransmitter systems [336–340], anti-anxiety/stress [341–344], sedation [343, 345–347], and anti-insomnia properties [100]. Its local anesthetic effects [348] were equivalent to procaine and menthol [349], and analgesic effects have been attributed to adenosine A_2A_ activity [350] and ionotropic glutamate receptors including AMPA, NMDA and kainate [351]. Morphine opioid usage in gastric banding surgical patients was significantly decreased following lavender inhalation vs. placebo, and this was attributed to the linalool concentration [352].

Beta-caryophyllene (β-caryophyllene) is found in spices and plants including cloves, cinnamon, black pepper, hops, rosemary, oregano, and basil. It has analgesic effects in inflammatory and neuropathic pain [353], and has potent anti-inflammatory effects [354–357], with local anesthetic properties [358]. Anti-inflammatory effects appear to occur via PGE-1 [359], with similar efficacy as indomethacin and etodolac [360, 361], and comparable to phenylbutazone [359, 360]. β-caryophyllene is a selective cannabinoid receptor 2 (CB2) agonist [362–364]. CB2 receptors have been implicated in anxiety and depression, and β-caryophyllene has shown anxiolytic and antidepressant effects [365].

Humulene (α-caryophyllene) is an isomer of β-caryophyllene and plays a role in many of the distinguishing characteristics between different cannabis strains. It is found in herbs and spices such as clove, basil, hops, sage, spearmint and ginseng, in addition to some vegetables and fruits. It has strong anti-inflammatory properties comparable to dexamethasone systemically, topically, and in allergic airway inflammation [354–356, 366, 367], as well as anti-nociceptive and analgesic properties [367].

Nerolidol (trans-nerolidol) is found in many herbs and spices including lavender, lemon grass, ginger, jasmine, tea tree, oranges, and present in orange and other citrus peels. It has anti-insomnia and sedative properties [368].

Alpha-bisabolol (α-bisabolol, bisabolol, levomenol) is produced by some flowers used in making tea, such as the chamomile flower. It has anti-inflammatory effects in the skin [369], as well as anti-nociceptive properties [370].

*Cannabis sativa* strains are generally described by patients as uplifting, energetic, creative, euphoria, spacey, cerebrally-focused effects, and better for day use, while *Cannabis indica* strains are typically described as calming, relaxing, sedative, full body effects such as “body buzz”, and better for night use. Research suggests these effects are not likely due purely to CBD:THC ratios, as there are no significant differences in CBD:THC ratios between Sativa and Indica strains. Rather these different subjective effects are likely due to varying ratios of major cannabinoids as well as minor cannabinoids, terpenes and probably additional phytochemicals [100, 371–374]. High CBD strains are Sativa or Indica strains that have been crossed with high CBD hemp strains (1:1 CBD:THC up to approximately 5:1 CBD:THC), while pure CBD strains (ratios of > 10:1 CBD:THC, which can be up to approximately 50:1 CBD:THC) are considered hemp strains. Most strains utilized today are Hybrids designed with standardized ratios of CBD, THC, other cannabinoids, and other compounds such as terpenes and flavonoids, targeting specific symptoms, responses, and end user effects.

Although not of statistical significance, there were some pattern use trends noted. The majority of patients across all pain groups including the positive ID Migraine™, headache as primary symptom, chronic pain, and arthritis groups all preferred Hybrid cannabis strains followed by Indica, Sativa, and higher CBD strains (1:1 CBD:THC, 3:1 CBD:THC) when patients with headache as primary symptom were included. However, when these patients were excluded, the arthritis group preferred Indica strains. When comparing headache and migraine to non-headache groups, Indica strains were preferred in the insomnia/sleep disorders group, Sativa strains in the mental health condition/PTSD group, and Hybrid strains were still preferred in the gastrointestinal disorder/Crohn’s Disease group. Perhaps the headache, chronic pain, and gastrointestinal disorder/Crohn’s groups preferred similar Hybrid strains due to underlying inflammatory pathophysiology. The positive ID Migraine™ and headache as primary symptom patients most commonly preferred the “OG Shark” Hybrid strain specifically, although this pattern was also noted in the chronic pain and arthritis groups, so was not unique to headache and migraine. This is a high THC/THCA, low CBD/CBDA strain with β-caryophyllene followed by β-myrcene as the predominant terpenes. This could reflect the potent analgesic, anti-inflammatory, and anti-emetic properties of THC, along with documented anti-inflammatory and analgesic properties of β-caryophyllene and β-myrcene. Given the prominent features of pain with nausea and vomiting in migraine headache, the fact that headache and migraine patients preferred a strain such as this, with its associated cannabinoid and terpene profile, would make sense given the known therapeutic effects of this cannabinoid and these terpenes. Furthermore, there were additional terpenes present in this strain of lower percentages, some of which also have analgesic and anti-inflammatory properties.

Substituting cannabis for alcohol, illicit drugs and/or prescription medications has been commonly observed in cross sectional surveys, suggesting a harm reduction role in the use of these substances, as well as implications for abstinence-based substance use treatment strategies [375–377]. The “opioid-sparing effect” of cannabinoids has been well described with extensive supporting evidence showing that combining cannabis with opiates decreases opiate dose requirements [166, 378]. CB1 receptors are 10 times more concentrated then mu-opioid receptors in the brain, and cannabinoid receptors co-localize with opioid receptors in many regions involved in pain pathways. This is suspected to contribute to synergistic augmentation of the analgesic opioid effects and decreased opioid dose requirements [8, 122–125, 166, 379–384], and studies have shown cannabis use did not affect blood levels of oxycodone or morphine [8, 166]. Cannabinoid receptor agonists increase endogenous opioid peptide release, and chronic THC use increases endogenous opioid precursor gene expression in supraspinal and spinal structures involved in pain perception [119, 126, 166, 379].

The synergistic effect of concomitant cannabis/cannabinoids and opioids in lowering both pain and opioid dose requirements without affecting serum opioid levels has been demonstrated prospectively [166]. A large meta-analysis showed that 17 of 19 pre-clinical studies provided good evidence of these synergistic effects from opioid and cannabinoid co-administration and that the median effective dose (ED50) of morphine administered with THC is 3.6 times lower than the ED50 of morphine alone, while the ED50 for codeine administered with THC was 9.5 times lower than the ED50 of codeine alone [378]. The combination of cannabis/cannabinoids and opioids appears to allow for opioid treatment at lower doses with fewer side effects, allowing easier detoxification and weaning due to lessening of tolerance and withdrawal from opiates, and rekindling of opiate analgesia after prior dosages have worn off [124]. Some pain specialists have suggested the use of medicinal cannabis treatment in addition to or in replacement of opiate treatments to help reduce overdose mortality and morbidity associated with opiate use [385]. Prospective studies have shown that chronic pain patients who use cannabis have improved pain and functional outcomes, and a significant reduction in opioid use [386], and medical cannabis use was associated with decreased opiate use, improvement in quality of life, and better side effect profile in a retrospective cross-sectional survey of chronic pain patients [387].

Notably, the most common prescription medications replaced by medicinal cannabis in this study were opiates/opioids in a large percentage within every pain group, up to 72.8% of patients in the chronic pain as primary illness group. Given the opioid epidemic, particularly in the United States, cannabis has been discussed as an option that may help in the opioid/opiate detoxification and weaning process and perhaps assist in combating the epidemic of opioid related death [377, 385, 388–390]. States with medicinal cannabis laws have been shown to have a 24.8% decreased annual opioid overdose mortality rate compared with states without medicinal cannabis laws. The association between medicinal cannabis law implementation and decrease in annual opioid overdose mortality strengthened over time to a decrease of 33.7% by year 5 [391].

The synergistic interactions between the phytocannabinoids, terpenes and other cannabis compounds resulting in various therapeutic benefits and responses have been termed the cannabis entourage effects [100, 170]. This synergy between the cannabinoids, terpenes, and other compounds leads to variable benefits, user effects, and strain characteristics. In addition, synergistic interactions between cannabis and opioid pathways may be a promising new weapon in the battle of the opioid epidemic. Further study is needed to determine optimal combinations for specific synergies and composition ratios of the cannabis constituents to best target different symptoms and diseases. Medicinal cannabis production has become a very sterile, scientific, standardized production process, and an emerging new industry. Similar to the broad category of anticonvulsants with many varieties targeting variable neurochemical pathways and channels with different responses and side effects, cannabis should also be thought of a broad category of medicine, of which further therapeutic delineations and disease targeting differentiations between strains is necessary.

There are multiple limitations to this study beginning with its survey design and inherent limitations. Many of the patients who reported headache as a primary symptom for which they were treating with medicinal cannabis, had also reported other diseases or symptoms that they were using medicinal cannabis for. So, some of the answers provided may not have been specific for only headache treatment, but potentially other symptoms or a combination of symptoms including headache. This could also influence reported preferred strains being used since some strains are used more commonly for some symptoms, while other strains may be used for other symptoms. There may be some inaccuracy of patient numbers within the different pain groups of chronic pain, arthritis, and headache. For example, some patients who reported chronic pain as the primary illness for which they were using medicinal cannabis did not specify their type of chronic pain further. It is unknown if some of these patients may have been treating chronic pain of arthritis or headache types, but reporting it as chronic pain, and therefore some of these patients may have been more accurately listed in a different more specific category. Variability in patients’ cannabis knowledge could potentially influence self-reporting accuracy. When documenting the preferred cannabis types and strains within each of the pain and non-pain groups, many patients did not provide an answer for their preferred type or strain. If a preferred cannabis type was not provided, but a preferred strain was provided, then their preferred type was presumed to correlate to their reported preferred strain, and counted as such. In addition, reported preferred cannabis types and strains sometimes did not correlate (reported strain did not fall under the correct reported type). Therefore, the preferred cannabis types and strains listed within each category, and their inferred potential benefits, may be inaccurate based on this inconsistent reporting by some patients, and the validity of the preferred cannabis type and strain data requires prospective validation.

## Conclusions

Chronic pain was the most common reason for use of medicinal cannabis, consistent with the statistics of most registries. Identifying differences in use patterns between migraine, headache, arthritis, and chronic pain syndromes may be helpful in optimizing crossbred cannabis strains, synergistic biochemical profiles, or dosing differences between these pain subsets. The majority of patients treating headache with medicinal cannabis were positive for migraine (88%) according to the ID Migraine™ questionnaire. This suggests that most headaches being treated with medicinal cannabis were likely of migrainous pathophysiology.

Hybrid cannabis strains were preferred across most pain groups. “OG Shark”, a high THC/THCA, low CBD/CBDA strain with β-caryophyllene followed by β-myrcene as the predominant terpenes, was the most preferred strain in the positive ID Migraine™ and headache as primary symptom groups. This could reflect the potent analgesic, anti-inflammatory, and anti-emetic properties of THC, along with documented anti-inflammatory and analgesic properties of β-caryophyllene and β-myrcene. Since migraines also involve nausea and vomiting, the potent antiemetic properties of THC may be a reason for this preference. Vaporizing or joint use were the primary methods of use across all groups, including migraine and headache, likely reflecting the need for a quick acting inhaled or non-orally ingested therapy in migraine attacks before severe pain and nausea/vomiting become prominent.

Most patients in the pain groups reported replacing prescription medications with medicinal cannabis, the most common of which were opiates/opioids across all pain groups. This is notable given the well-described “opioid-sparing effect” of cannabinoids and growing abundance of literature suggesting that cannabis may help in weaning from these medications and perhaps providing a means of combating the opioid epidemic. There are several limitations to the data in this study, and these results require further confirmation with more sophisticated prospective study methods. However, these results may provide early insight and a framework for direction into optimizing crossbred cannabis strains, synergistic biochemical profiles, dosing, and patterns of use that may be of clinical benefit in the treatment of headache and migraine, as well as other chronic pain syndromes.

## Abbreviations

2-AG: 2-arachidonoylglycerol
5-HT: 5-hydroxytryptamine (serotonin)
AEA: N-arachidonoylethanolamine (anandamide)
AMPA: α-amino-3-hydroxy-5-methyl-4-isoxazolepropionic acid
CB1: Cannabinoid 1 receptor
CB2: Cannabinoid 2 receptor
CB3: Cannabinoid 3 receptor
CBD: Cannabidiol
CBDA: Cannabidiolic acid
CGRP: Calcitonin gene related peptide
CML: Chronic myeloid leukemia
CMR1: Cold and menthol receptor 1
COX2: Cyclooxygenase-2
ED50: Median effective dose
FAAH: Fatty acid amide hydrolase
FDA: Federal drug administration
GABA: Gamma-aminobutyric acid
GPR55: G protein-coupled receptor 55
NMDA: N-methyl-d-aspartate
NNT: Number needed to treat
NSAID: Non-steroidal anti-inflammatory drug
PGE-1: Prostaglandin E1
PPAR: Peroxisome proliferator-activated receptors
PTSD: Post-Traumatic Stress Disorder
THC: Δ9-Tetrahydrocannabinol
THCA: Tetrahydrocannabinolic acid
TRPA: Transient receptor potential cation channel, subfamily A
TRPM: Transient receptor potential cation channel, subfamily M
TRPV: Transient receptor potential cation channel subfamily V
WADA: World Anti-Doping Agency
